# 130. Design and Preclinical Characterization of SER-155, an Investigational Cultivated Microbiome Therapeutic to Restore Colonization Resistance and Prevent Infection in Patients Undergoing Hematopoietic Stem Cell Transplantation

**DOI:** 10.1093/ofid/ofab466.130

**Published:** 2021-12-04

**Authors:** Elizabeth Halvorsen, Marin Vulic, Edward J O’Brien, Jessica Byrant, Mary-Jane Lombardo, Christopher Ford, Matt Henn

**Affiliations:** 1 Seres Therapeutics, Cambridge, MA; 2 Seres Therapeutics, Inc, Cambridge, Massachusetts; 3 Seres Therapeutics, Inc., Cambridge, MA

## Abstract

**Background:**

During allogeneic hematopoietic stem cell transplant (HSCT), the diversity and stability of the GI microbiome is disrupted, increasing the risk of domination by pathogens associated with bacteremia, aGvHD, and mortality. SER-155 is an investigational, oral microbiome therapeutic composed of cultivated spores and vegetative bacterial strains rationally designed to reduce the risk of bacteremia and aGvHD in HSCT recipients by decolonizing potential pathogens and restoring GI colonization resistance. SER-155 was evaluated *in vitro* for key pharmacological properties associated with colonization resistance, and *in vivo* to assess its ability to restore colonization resistance by reducing *Enterococcus* and *Enterobacteriaceae* carriage.

**Methods:**

The design of SER-155 leveraged genomic data from interventional and observational human datasets to include taxa associated with reduced risk of infection and aGvHD in HSCT. Strains of interest were phenotyped, and over 50 candidate consortia containing different combinations of over 150 species were designed and tested *in vitro* and *in vivo*. *In vivo*, candidate compositions were evaluated in mouse models of vancomycin-resistant *Enterococcus faecium* (VRE) and carbapenem-resistant *Klebsiella pneumoniae* (CRE) colonization.

**Results:**

Oral administration of SER-155 led to a 2-3 Log_10_ reduction in VRE and CRE titers compared to untreated mice (Figure 1). *In vitro*, the carbon source utilization profile of VRE, CRE, and SER-155 strains were assessed using a panel of 85 carbon sources. All 56 carbon sources used by CRE or VRE for anaerobic growth were also utilized by SER-155 strains, supporting a model in which nutrient competition may contribute to reducing CRE and VRE carriage and restoring colonization resistance.

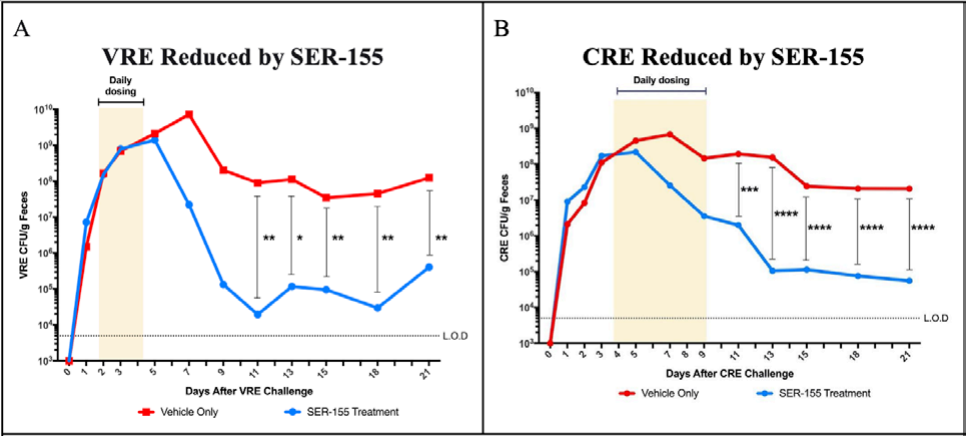

Figure 1. SER-155 Efficacy in Mouse Models of VRE and CRE Colonization.

The titers of VRE or CRE were quantified in fecal pellets by plating on selective agar at the indicated time-points. The median A) VRE and B) CRE CFU per gram of feces was calculated for each group and plotted on the line graph (n=6-10 per group). L.O.D., limit of detection. Data were analyzed using the Mann-Whitney t-test and significance was determined as a p-value of p< 0.05*, p<0.01**, p<0.001***, p<0.0001****.

**Conclusion:**

SER-155 is an investigational cultivated microbiome therapeutic intended to reduce the risk of infection by engrafting human-commensal bacterial strains in adults undergoing allogeneic HSCT. Preclinical assessments *in vitro* and *in vivo* support the ability of SER-155 to reduce VRE and CRE carriage and restore colonization resistance in the gut. A Phase 1b study evaluating SER-155 in allogeneic HSCT patients is being planned.

**Disclosures:**

**Elizabeth Halvorsen, PhD**, **Seres Therapeutics** (Employee, Shareholder) **Marin Vulic, PhD**, **Seres Therapeutics** (Employee) **Edward J. O’Brien, PhD**, **Seres Therapeutics** (Employee, Shareholder) **Jessica Byrant, PhD**, **Seres Therapeutics** (Employee, Shareholder) **Mary-Jane Lombardo, PhD**, **Seres Therapeutics** (Employee, Shareholder) **Christopher Ford, PhD**, **Seres Therapeutics** (Employee, Shareholder) **Matt Henn, PhD**, **Seres Therapeutics** (Employee, Shareholder)

